# Educational

**Published:** 1892-10

**Authors:** 


### EDITORIAL.

### Educational.

The time has arrived when our dental colleges open their doors for the reception of students—the time when another course of educational work begins; an interesting and important time for the student and the teacher as well; a time of interest to all members of the dental profession who are solicitous for its welfare.

The present is a time of especial interest in college matters; practically most of our colleges enter the year under new auspices, viz.: the adoption of three full courses of not less than five months each for graduation. This, however, came into force last year, but more fully now, because the last of the two years' students will disappear with this term, and a far less number of these will be present than last year; and doubtless most of the colleges will make higher requirements in regard to preliminary education. The last term before the three year requirements and while a very mild preliminary examination was required by most colleges there was a large influx of students, many of whom were influenced by the lower requirements. The free discussion on the subject of dental education had in the American

Dental Association, in the Southern Dental Association, in the Association of Dental Faculties, and the Board of Dental Examiners is a stimulus to the colleges to raise their requirements and to exercise more care in the reception of students as well as in their education and graduation.

The probability is that for the present year nearly if not quite all the dental colleges will have smaller classes than for the last year or two, and indeed there will not probably in the near future be so great a rush of students into our colleges as in the last two years. One result of this great increase of students has been the organization of new colleges, some ten or twelve of which have been inaugurated during the last two years; this was unprecedented and quite beyond the normal growth; whether these will all continue their existence is a question about which, of course, there is much difference of opinion.

The elevation of requirements in dental education has elicited the attention of the better educated and more cultured class of students. This progress should not, and will not stop at this stage. The beneficial results of the progress already made will be so apparent to the profession as to meet its entire approval and its demand that this progress shall continue. How very desirable it is, that all our colleges should fully respond to the trend of the times, in this respect, we need here hardly attempt to specify. Large classes and corresponding emoluments seem to have been the chief aim of some. Faculties, however, are not wholly responsible for the entrance of unworthy students; great care and vigilance in many instances are insufficient to prevent this; faculties of dental colleges may be and are imposed upon, as well as any other class of people, but such an occurrence should be reduced to the smallest possible degree. It would be well to fully accept only those who can establish their claims for acceptance to an undoubted degree, and let those who cannot do this be held in abeyance or conditioned until time shall reveal their true character. Medical and dental faculties should exercise the greatest care and discrimination in regard to the admission of those whom they admit to their classes.

We trust and believe that the present year will show a far better and more earnest class of students in our colleges than heretofore. The increase of time for graduation will be attractive only to the student of the greater earnestness, determination, and industry; the hasty slip-shod student is far less likely to enter upon this than upon the former short term, and besides, the greater discrimination in admission will serve in a large measure to discourage the poor student.

The profession will look with great interest to the outcome of the present year's work in our colleges, and doubtless will expect a marked improvement in many particulars.

